# SwinChange-DE: A Detail-Preserving Hierarchical Transformer Framework for High-Resolution Remote Sensing Image Change Detection

**DOI:** 10.3390/s26206571

**Published:** 2026-10-16

**Authors:** Tingwei Hu, Wanwen He, Jiaxin Yang, Jing Wu, Congqin Yi, Zhenhua Wang

**Affiliations:** 1 College of Information Technology, Shanghai Ocean University, Shanghai 201306, China; 2 Shandong Provincial Institute of Territorial Space Data and Remote Sensing Technology, Shandong Provincial Center for Dynamic Monitoring and Surveillance of Sea Areas, Jinan 250014, China

**Keywords:** remote sensing change detection, Swin Transformer, multi-scale feature fusion, boundary refinement, small-scale change detection

## Abstract

High-resolution remote sensing image change detection plays an important role in urban monitoring, disaster assessment, and land-cover analysis. However, complex spatial structures and diverse object scales make accurate change localization challenging, while progressive downsampling can obscure object boundaries and small changes. To address these challenges, this paper proposes SwinChange-DE, a detail-preserving hierarchical Transformer framework for high-resolution remote sensing image change detection. A Swin-inspired encoder extracts spatial–semantic representations at multiple resolutions through efficient contextual modeling. To bridge the semantic gap across feature levels, an adaptive fusion strategy refines shallow features under deep semantic guidance before aggregation. A detail enhancement module further leverages fused change information to guide the integration of high-resolution features from both temporal images during decoding, supporting boundary recovery and the detection of small-scale changes. Together, these components connect contextual representation, cross-scale alignment, and precise change localization within a unified framework. Extensive experiments were conducted on the LEVIR-CD+ and WHU-CD benchmarks. SwinChange-DE achieves F1-scores of 87.66% and 87.43%, respectively, outperforming the best-performing competing methods by 0.25% and 0.93% in F1-score. The results demonstrate the effectiveness of the proposed framework in improving boundary delineation and recovering subtle changes in high-resolution remote sensing imagery.

## 1. Introduction

Remote sensing image change detection (CD) aims to identify significant variations between multi-temporal remote sensing observations and has become an essential technique for a wide range of applications, including urban expansion monitoring, land-cover mapping, environmental assessment, and disaster response [1,2]. With the rapid development of high-resolution remote sensing satellites, increasingly detailed spatial information has become available, enabling the detection of fine-scale object changes. However, the complex spatial structures, diverse object scales, and spectral variations in high-resolution remote sensing imagery introduce substantial challenges for accurate change detection, particularly in preserving object boundaries and identifying small-scale changes.

Traditional change detection approaches mainly rely on manually designed features and statistical analysis methods, such as image differencing, change vector analysis, and threshold-based techniques [3,4,5]. Although these methods are computationally efficient, their performance heavily depends on handcrafted feature selection and predefined decision rules. Consequently, they often struggle to handle complex scenes with diverse spatial patterns, illumination variations, and heterogeneous backgrounds, limiting their applicability to high-resolution remote sensing imagery [6,7,8,9].

With the advancement of deep learning, convolutional neural networks (CNNs) have achieved remarkable progress in remote sensing change detection by automatically learning hierarchical feature representations from data [10,11]. Compared with traditional approaches, CNN-based methods can effectively extract discriminative spatial features and improve robustness under complex scenarios. Encoder–decoder architectures, such as U-Net and its variants, have been widely adopted because they can integrate high-level semantic information with low-level spatial details through skip connections [12,13,14].

However, CNN-based approaches are inherently constrained by the local receptive field of convolution operations, making it difficult to capture long-range dependencies and global contextual relationships in complex remote sensing scenes [15]. This limitation becomes particularly evident in high-resolution imagery, where objects often exhibit large spatial variations and contextual interactions. Although deeper networks and larger convolution kernels can partially enlarge the receptive field, they usually introduce increased computational complexity and may still fail to establish effective global representations.

To overcome the limitation of insufficient long-range dependency modeling in CNN-based approaches, Transformer architectures have been introduced into remote sensing change detection. By employing self-attention mechanisms, Transformer-based methods can establish global interactions among image features beyond local receptive fields, providing stronger capability for modeling complex spatial relationships [16]. Early Transformer-based change detection methods demonstrated improved performance by leveraging global contextual information and more flexible feature representation strategies.

Recently, hierarchical Transformer architectures, such as Swin Transformer, have further improved the efficiency of Transformer models by introducing shifted-window attention and multi-scale feature representation mechanisms [17]. These approaches effectively combine local feature extraction with global dependency modeling, making them suitable for high-resolution remote sensing image analysis.

However, progressive spatial downsampling in hierarchical Transformer encoders can weaken the spatial cues required for accurate change localization. From an image-processing perspective, object boundaries and narrow structures involve rapid spatial variations and therefore contain high-spatial-frequency information that is sensitive to resolution reduction. As the feature maps become coarser, each spatial position represents a larger image region, making small changed objects less distinguishable from their surroundings. When neighboring spatial representations are aggregated and compressed into fewer tokens, subtle differences between foreground and background may also be attenuated. Although some local information can be retained in feature channels, precise spatial relationships are not necessarily preserved. Subsequent upsampling increases the feature-map resolution but cannot, by itself, reliably reconstruct information that is no longer encoded. These effects can contribute to blurred boundaries, incomplete change regions, and missed small changes, motivating the incorporation of high-resolution spatial cues into the decoding process.

To address this issue, existing methods introduce multi-scale feature fusion and attention mechanisms to combine information across resolution levels and emphasize informative regions [18,19,20]. Nevertheless, aggregating shallow and deep features does not necessarily resolve their semantic discrepancy, and direct fusion may retain irrelevant responses. The resulting representations may also provide insufficient spatial information for reconstructing complete change regions during decoding [21,22,23]. These limitations motivate the joint design of semantic-guided feature alignment and explicit detail recovery.

Accordingly, we propose SwinChange-DE, a detail-preserving hierarchical Transformer framework for high-resolution remote sensing image change detection. Built upon ChangeFormer, the framework uses a Swin-inspired encoder for multi-level contextual representation. Different from conventional feature fusion and decoder refinement strategies that directly aggregate multi-level representations, SwinChange-DE introduces a task-oriented refinement process that integrates semantic-guided cross-scale alignment and detail-preserving decoding. Specifically, the Gate-Enhanced Self-Modulation Feature Aggregation (GE-SMFA) module refines shallow features under deep semantic guidance before feature aggregation, while the Change Detail Enhancement Decoder Module (CDDEM) uses fused change information to guide the incorporation of high-resolution features from both temporal images into the decoding process. Together, these components form a semantic-guided progressive refinement pipeline, where contextual representation learning, cross-scale alignment, and detail recovery are sequentially optimized for accurate change localization.

The main contributions of this work are summarized as follows: 

(1) A detail-preserving hierarchical Transformer framework is proposed for high-resolution remote sensing image change detection. By incorporating a Swin-inspired hierarchical encoder into the ChangeFormer architecture, the proposed framework enhances multi-scale spatial–semantic representation learning through efficient window-based contextual modeling, providing a more suitable feature representation for fine-grained change analysis.

(2) A semantic-guided cross-scale feature alignment strategy is developed to improve the interaction between shallow spatial details and deep semantic representations. Different from direct multi-level feature aggregation, the proposed strategy progressively refines shallow features using higher-level change semantics before feature fusion, reducing feature inconsistency across scales and improving change representation in complex scenes.

(3) A detail-preserving decoder refinement strategy is introduced to enhance spatial detail recovery during decoding. Instead of directly combining shallow and deep features, CDDEM uses high-level change semantics to guide the selection and integration of bi-temporal high-resolution features, thereby reducing background interference, improving boundary localization, and preserving complete change structures.

## 2. Methodology

**Figure 1:**
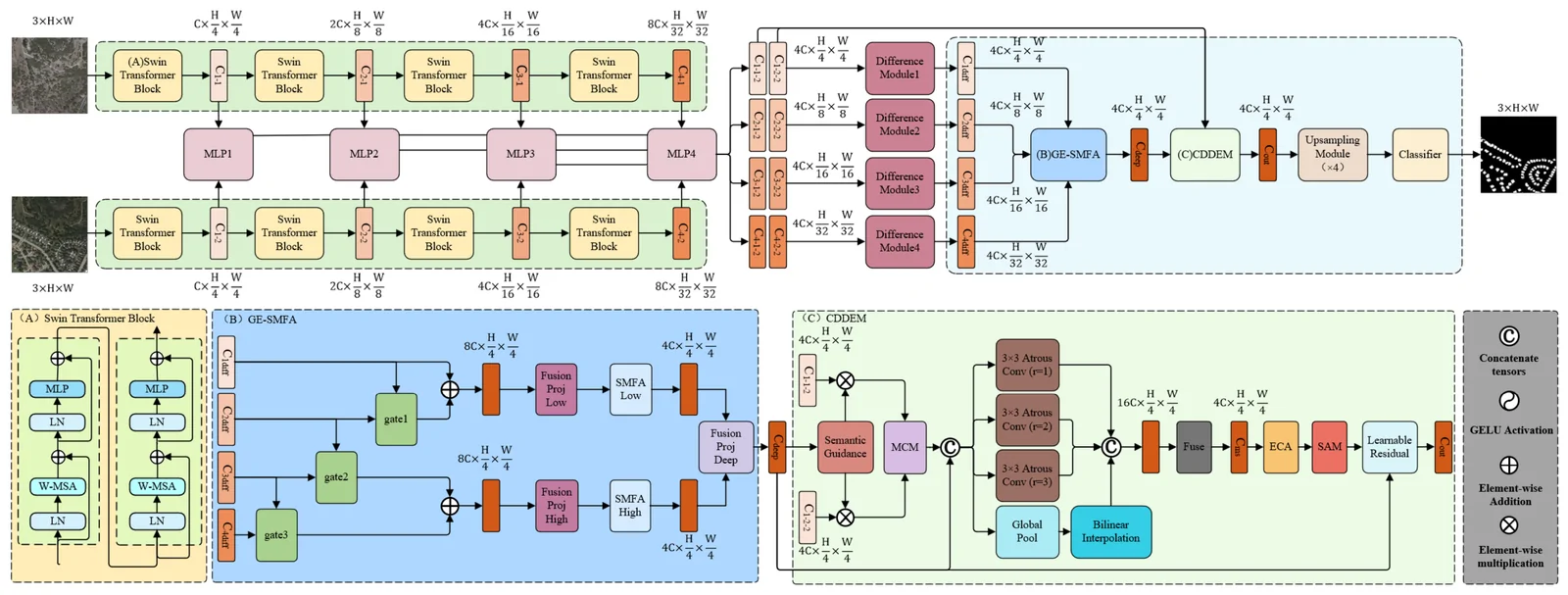
Architecture of the SwinChange-DE framework for high-resolution remote sensing image change detection. The framework consists of three core components: (**A**) Swin-inspired hierarchical encoder for hierarchical spatial–semantic representation learning, (**B**) GE-SMFA for cross-scale semantic alignment, and (**C**) CDDEM for spatial detail recovery and boundary refinement during decoding.

To address the challenges of preserving fine-grained spatial details while maintaining strong semantic representation in high-resolution remote sensing change detection, we propose SwinChange-DE, a detail-preserving hierarchical Transformer framework built upon the ChangeFormer architecture [24]. As illustrated in Figure 1, the proposed framework consists of three complementary components designed to progressively enhance spatial–semantic representation during the encoding, feature interaction, and decoding stages.

Specifically, the Swin-inspired hierarchical encoder is employed to construct hierarchical spatial–semantic representations by capturing multi-level contextual information with efficient attention modeling. Subsequently, the GE-SMFA module performs cross-scale feature alignment by refining shallow spatial features under deep semantic guidance and establishing effective global–local feature interaction. Finally, the CDDEM module focuses on spatial detail recovery during decoding by integrating high-resolution shallow features with deep semantic representations, thereby improving boundary delineation and fine-grained change localization.

Through the collaborative optimization of hierarchical representation learning, cross-scale feature alignment, and spatial detail recovery, SwinChange-DE achieves a better balance between semantic understanding and spatial detail preservation for high-resolution remote sensing image change detection.

### 2.1. Swin-Inspired Hierarchical Encoder for Hierarchical Spatial–Semantic Representation

High-resolution remote sensing change detection requires effective feature representations that can simultaneously characterize spatial details and contextual semantics. However, complex backgrounds, heterogeneous land-cover patterns, and significant variations in object scales make it difficult for conventional feature extraction methods to establish comprehensive spatial–semantic representations. Although Transformer-based architectures, such as ChangeFormer, have demonstrated strong contextual modeling capability through self-attention mechanisms, processing high-resolution feature maps can still incur considerable computational costs. Therefore, an efficient hierarchical representation strategy is essential for balancing contextual modeling capability and computational efficiency in high-resolution change detection.

To obtain hierarchical spatial–semantic representations, SwinChange-DE constructs a Swin-inspired hierarchical encoder by replacing the original Transformer blocks in the ChangeFormer encoder with Swin Transformer blocks [25], while retaining overlapping convolutional embeddings and progressive spatial downsampling. This constitutes a modified encoder rather than a standard Swin Transformer backbone. Across the four stages, the block depths, channel dimensions, and attention head numbers are set to [2,2,6,2], [64,128,256,512], and [1,2,4,8], respectively. Each block employs window-based multi-head self-attention within 8 × 8 non-overlapping windows, with no window shifting.

The embedding layers at all four stages are implemented using the OverlapPatchEmbed module, with 7 × 7 convolutional kernels, strides of [4,2,2,2], and zero padding of 3 pixels on each side, followed by layer normalization. Specifically, Stage 1 reduces the input resolution from *H* × *W* to $\frac{H}{4}\times \frac{W}{4}$, generating the initial bi-temporal feature maps $C_{1\_1},C_{1\_2}\in R^{64\times \frac{H}{4}\times \frac{W}{4}}$. Subsequent stages progressively reduce spatial resolution and integrate information across neighboring spatial regions, while window-based self-attention within each stage captures local spatial relationships. This hierarchical design combines local spatial modeling with progressively broader contextual representations to support efficient feature extraction.

Compared with the standard Multi-Head Self-Attention (MSA) used in conventional Transformers [26], the Window-based Multi-Head Self-Attention (W-MSA) in the Swin Transformer substantially reduces computational overhead for high-resolution remote sensing imagery [27]. The computational complexities of MSA and W-MSA are defined as

*Ω*(*MSA*) = 4*hwC*^2^ + 2(*hw*)^2^*C* (1)

*Ω*(*W*-*MSA*) = 4*hwC*^2^ + 2*M*^2^*hwC* (2)

where *h* and *w* denote the height and width of the feature map, *C* represents the number of channels, and M indicates the window size. Compared with global self-attention, W-MSA limits the attention operation within local windows, reducing the quadratic computational cost associated with spatial resolution while preserving effective local-context modeling.

Furthermore, the four-stage hierarchical architecture enables multi-level spatial–semantic representation learning for change detection. Specifically:

- Shallow stages (Stages 1 and Stage 2) generate high-resolution feature maps $C_{1\_1},\;C_{1\_2}\in R^{C\times \frac{H}{4}\times \frac{W}{4}}$ and $C_{2\_1},\;C_{2\_2,}\in R^{2C\times \frac{H}{8}\times \frac{W}{8}}$, respectively. These features retain rich spatial information, including texture, edges, and local geometric structures, which are essential for accurate boundary localization and the identification of small-scale changes, such as minor building modifications and newly constructed facilities.
- Deep stages (Stages 3 and 4) further compress spatial resolution to $\frac{H}{16}\times \frac{W}{16}$ and $\frac{H}{32}\times \frac{W}{32}$, generating higher-level semantic representations *C*_3_1_, *C*_3_2_ and ${C_{4\_1},\;C}_{4\_2}$. These deep features provide stronger contextual understanding and are effective for capturing large-scale structural changes, such as land-use transformation, regional expansion, and major infrastructure modifications.

Through this hierarchical encoding process, shallow features provide accurate spatial localization, while deep features provide semantic abstraction and global contextual information. The complementary spatial–semantic representations establish a robust foundation for subsequent GE-SMFA and CDDEM modules, enabling effective cross-scale semantic interaction and spatial detail recovery in SwinChange-DE.

### 2.2. GE-SMFA: Gate-Enhanced Self-Modulation Feature Aggregation

A key challenge in high-resolution remote sensing change detection is how to effectively integrate high-resolution spatial details with deep semantic representations. Although hierarchical Transformer architectures generate multi-level features with complementary information, the semantic discrepancy between shallow spatial features and deep semantic features may hinder effective feature interaction. The original ChangeFormer mainly relies on linear projection and channel-wise concatenation for feature fusion. While this strategy is simple and computationally efficient, it lacks explicit modeling of spatial dependencies and semantic relationships across different feature levels. As a result, directly combining heterogeneous features may introduce irrelevant responses, weaken boundary representation, and reduce the detection capability for small-scale changes.

To address this issue, we propose the Gate-Enhanced Self-Modulation Feature Aggregation (GE-SMFA). GE-SMFA is developed based on the Self-Modulation Feature Aggregation (SMFA) [28] and extends the original SMFA by introducing a cross-scale progressive gating mechanism specifically designed for semantic-guided feature alignment in change detection. Different from conventional feature aggregation strategies that directly combine heterogeneous features from different levels, GE-SMFA introduces a semantic-guided refinement stage before feature aggregation. By progressively transferring high-level change semantics to shallow features, GE-SMFA aims to reduce cross-scale feature inconsistency and provide more reliable representations for subsequent multi-scale interaction. Subsequently, the refined features are processed by the SMFA module to establish complementary global–local feature interaction and generate more discriminative multi-scale representations.

GE-SMFA consists of two sequential components: (1) Cross-Scale Progressive Gate for semantic-guided feature refinement and (2) SMFA for multi-scale feature interaction.

**(1) Cross-Scale Progressive Gate**

The Cross-Scale Progressive Gate aims to reduce the semantic discrepancy between shallow spatial features and deep semantic representations before feature fusion [29]. Shallow features contain rich spatial details, such as edges and local geometric structures, but are also sensitive to background interference and spectral variations. In contrast, deep features provide stronger semantic abstraction but contain limited spatial information due to hierarchical feature extraction.

Therefore, a top-down guidance strategy is introduced to progressively refine shallow features using deep semantic information. To implement this strategy, the bi-temporal encoder features at each stage are first projected to a common channel dimension of 4*C* through stage-specific linear layers, and the projected features from the two temporal images are concatenated and processed by convolutional difference modules to construct change features. During progressive guidance, each coarser-scale change feature is bilinearly upsampled to match the spatial dimensions of the adjacent finer-scale feature. Inspired by spatial gating mechanisms [30], the proposed gate generates spatial modulation responses from the resized coarser-scale features to selectively enhance finer-scale change responses.

Furthermore, the residual attention mechanism [31] is adopted to maintain stable feature propagation during feature refinement. Through this semantic-guided filtering process, the consistency between shallow and deep representations is improved, providing more reliable features for subsequent multi-scale interaction.

**(2) SMFA: Multi-Scale Feature Fusion for Fine-Grained Change Detection**

**Figure 2:**
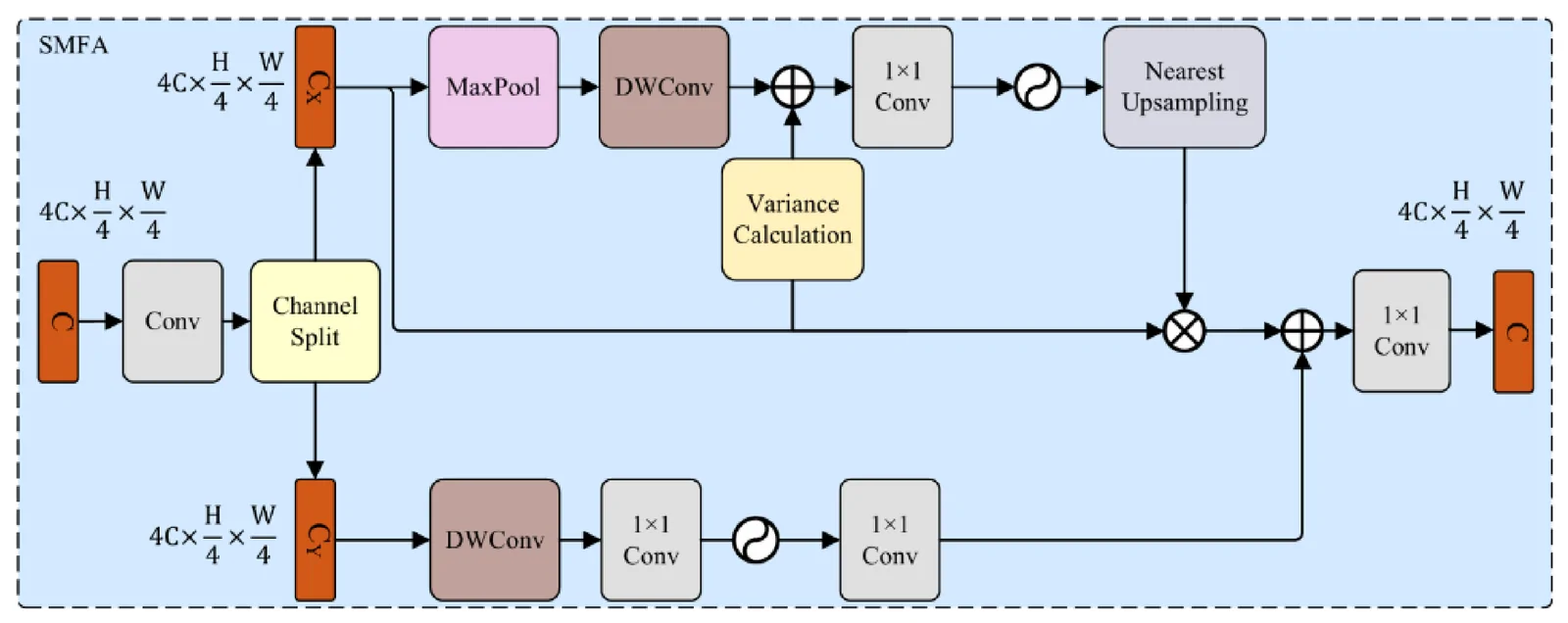
Architecture of the SMFA component, consisting of the EASA branch for contextual semantic modeling and the LDE branch for local detail enhancement.

After semantic-guided refinement by the Cross-Scale Progressive Gate, the change features from Stages 2–4 are bilinearly upsampled to $\frac{H}{4}\times \frac{W}{4}$ to match the Stage 1 feature. The aligned features are then grouped into lower-stage and higher-stage pairs for SMFA aggregation. As illustrated in Figure 2, the SMFA module consists of two complementary branches: the Efficient Approximation of Self-Attention (EASA) branch for contextual semantic modeling and the Local Detail Estimation (LDE) branch for local spatial detail enhancement.

The two branches focus on different aspects of feature representation. The EASA branch captures long-range contextual relationships with reduced computational complexity, while the LDE branch enhances local spatial structures and fine-grained details. By integrating these complementary representations, SMFA improves spatial–semantic feature interaction and strengthens the representation capability for complex remote sensing scenes.

**EASA Branch: Contextual Semantic Modeling**

The EASA branch is designed to capture global contextual information efficiently. It first employs adaptive max pooling combined with 3 × 3 depth-wise convolutions to extract low-frequency structural information. Meanwhile, the global variance of feature responses is calculated to characterize feature distribution variations across different spatial regions.

These structural and statistical representations are adaptively integrated through learnable parameters to generate contextual modulation responses. The generated responses are subsequently applied to the input features through element-wise multiplication, enabling adaptive enhancement of important semantic regions. This process improves global contextual modeling capability while maintaining computational efficiency for high-resolution remote sensing imagery.

**LDE Branch: Local Detail Enhancement**

Complementary to the EASA branch, the LDE branch focuses on local detail estimation. It employs a depth-wise convolution followed by two 1 × 1 convolutions with a hidden GELU activation to capture and refine local spatial correlations.

By strengthening local spatial responses, the LDE branch enhances object boundaries and small-scale change patterns that may be weakened during hierarchical feature extraction. The generated local representations provide detailed spatial cues complementary to the semantic information captured by the EASA branch.

Finally, the outputs from the EASA and LDE branches are integrated through element-wise addition, followed by a 1 × 1 convolution for feature alignment. Through global–local feature interaction, SMFA effectively reduces the semantic discrepancy across feature levels and generates more discriminative multi-scale representations.

Overall, GE-SMFA performs semantic-guided feature refinement before feature fusion and establishes effective interaction between shallow spatial details and deep semantic representations. Its output, $C_{\mathrm{deep}}\in R^{4C\times \frac{H}{4}\times \frac{W}{4}}$, represents the fused multi-scale change features and provides semantic guidance for subsequent CDDEM-based detail recovery and final change prediction.

### 2.3. CDDEM: Detail Recovery and Boundary Refinement in the Decoder

Although hierarchical Transformer encoders provide strong semantic representations, progressive spatial downsampling can weaken fine spatial details and boundary information [32]. Decoding from aggregated multi-level representations may therefore provide insufficient access to the fine-grained cues needed for accurate boundary localization and small-scale change detection. To address this limitation, we introduce the Change Detail Enhancement Decoder Module (CDDEM), which provides an explicit pathway for incorporating high-resolution shallow features from both temporal images into the decoding process. These features supplement the fused multi-scale change representation with spatial details that may be attenuated during hierarchical encoding and feature aggregation.

The methodological distinction of CDDEM lies in the semantic-conditioned processing order of detail recovery. Instead of directly concatenating shallow and deep features, CDDEM first filters high-resolution features using change-semantic guidance and subsequently constructs detail-based change representations. Shallow features contain useful spatial structures but also background textures and appearance variations unrelated to target changes. CDDEM therefore first uses the fused change features to generate weights and applies the same weights to the shallow features from both temporal images. Edge differences and feature-level interactions are constructed only after this semantic-guided filtering. This processing order explicitly conditions the construction of detail-based change representations on the aggregated change semantics, rather than relying solely on direct concatenation of shallow and deep features.

CDDEM comprises three sequential stages, each addressing a specific requirement of this detail recovery pathway. (1) Semantic-Guided Adaptive Filtering uses the shared modulation map to selectively attenuate shallow-feature responses, with the aim of reducing irrelevant information before temporal interaction. (2) Multi-dimensional Change Interaction and Context Aggregation constructs edge differences, absolute differences, element-wise products, and sums from the filtered features and combines them with the change-semantic representation through ASPP to capture complementary temporal interactions and spatial context. (3) Attention-guided Detail Refinement and Semantic Preservation applies ECA and SAM to recalibrate the aggregated features, followed by a learnable residual fusion that retains a direct contribution from the change-semantic representation.

**(1) Semantic-Guided Adaptive Filtering**

High-resolution shallow features contain abundant spatial information, including edges, textures, and local geometric structures. However, these features are also susceptible to background interference, spectral variation, and pseudo-change responses. Therefore, directly introducing shallow features into the decoder may degrade the quality of change representation.

To address this issue, CDDEM introduces a semantic-guided adaptive filtering strategy, where deep semantic features are utilized as guidance information to refine shallow features. Specifically, the shallow features $C_{1\_1\_2},{\;C}_{1\_2\_2}\in R^{4C\times \frac{H}{4}\times \frac{W}{4}}$ are adaptively filtered by a spatial-channel weight map generated from the deep feature *C_deep_*. The weight map is obtained through depth-wise convolution, 1 × 1 convolution, batch normalization, and Sigmoid activation:

(3) $$W=\sigma \left(\mathrm{BN}({\mathrm{Conv}}_{1\times 1}({\mathrm{DConv}}_{3\times 3}(C_{\mathrm{deep}})))\right)\in R^{4C\times \frac{H}{4}\times \frac{W}{4}}$$

where *W* represents the adaptive modulation weight. The refined shallow features are then obtained through element-wise modulation:

(4) $${\hat{C}}_{1\_i\_2}=C_{1\_i\_2}⊙W$$

This semantic-guided filtering process suppresses irrelevant background responses while preserving discriminative spatial structures, providing cleaner detail information for subsequent change interaction.

**(2) Multi-dimensional Change Interaction and Context Aggregation**

After adaptive filtering, CDDEM further explores complementary change information from the bi-temporal shallow features. Since different forms of feature interactions capture different aspects of temporal variation, we explicitly construct multiple change representations, including edge difference, absolute difference, element-wise product, and element-wise sum.

First, spatial edge information is extracted using fixed Sobel operators to characterize boundary variations. For each temporal feature, the edge representation is calculated as follows:

(5) $$E_{i}=\sqrt{({\mathrm{Sobel}}_{x}\times {\hat{C}}_{1\_i\_2}{)}^{2}+({\mathrm{Sobel}}_{y}\times {\hat{C}}_{1\_i\_2}{)}^{2}+\epsilon }\in R^{4C\times \frac{H}{4}\times \frac{W}{4}}$$

The edge difference between two temporal observations is then obtained as follows:

*C_edge_* = |*E*_1_ − *E*_2_| (6)

In addition to boundary variation, CDDEM computes the absolute difference *C_diff_*, element-wise product *C_prod_*, and element-wise sum *C_sum_* between the bi-temporal features to capture complementary nonlinear change relationships.

**Figure 3:**
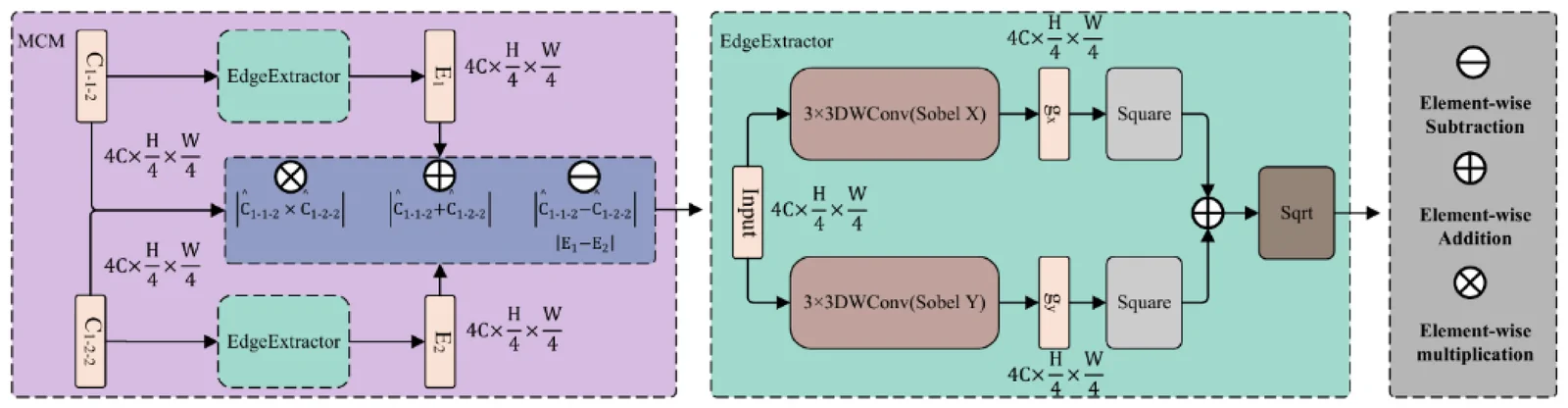
Detailed structure of the multi-dimensional change interaction module (MCM) in CDDEM.

As illustrated in Figure 3, the multi-dimensional change interaction module (MCM) extracts complementary temporal features from the filtered bi-temporal representations, including edge differences and feature-level interactions. These features are concatenated with the deep semantic feature *C_deep_* along the channel dimension to combine spatial details and semantic context.

To further aggregate multi-scale contextual information, the concatenated representation is processed through a lightweight Atrous Spatial Pyramid Pooling (ASPP) module [33] with dilation rates of 1, 2, and 3, together with a global adaptive average pooling branch. The outputs of different branches are fused through a 3 × 3 convolution to generate the unified multi-scale representation *C_ms_*.

**(3) Attention-guided Detail Refinement and Semantic Preservation**

Although multi-dimensional interaction enhances change representation, redundant responses may still exist after feature aggregation. Therefore, CDDEM introduces a sequential attention refinement strategy to further enhance informative spatial and channel responses.

Inspired by the sequential attention design in HCF-Net [34], the aggregated feature *C_ms_* is first processed by Efficient Channel Attention (ECA) [35], which adaptively emphasizes important feature channels without dimensionality reduction. Subsequently, the Spatial Attention Module (SAM) [36] is applied to enhance pixel-level spatial responses and improve boundary localization.

To dynamically balance the contribution of refined detail features and deep semantic information, a learnable gated residual connection [37] is introduced:

(7) $$C_{\mathrm{out}}=\mathrm{SAM}\left(\mathrm{ECA}\left(C_{\mathrm{ms}}\right)\right)+\alpha \cdot C_{\mathrm{deep}}\in R^{4C\times \frac{H}{4}\times \frac{W}{4}}$$

where *α* denotes a learnable parameter controlling the contribution of deep semantic information. This gated residual strategy enables adaptive integration of enhanced spatial details and semantic representations, improving structural consistency during decoding.

Through these operations, CDDEM effectively restores spatial information weakened during hierarchical feature extraction and establishes a better balance between detailed structures and semantic understanding. By combining semantic-guided filtering, multi-dimensional change interaction, and attention-based refinement, CDDEM improves boundary delineation, suppresses pseudo-change responses, and enhances the detection capability of subtle spatial variations in complex high-resolution remote sensing scenes. Unlike conventional decoder strategies that directly concatenate multi-scale features or independently apply attention refinement, CDDEM adopts a semantic-guided progressive refinement pipeline, where spatial details are selectively recovered under high-level change constraints.

## 3. Experimental Data and Setup

### 3.1. Experimental Data

The experiments were conducted on two widely used high-resolution remote sensing change detection benchmarks: WHU-CD [38] and LEVIR-CD+ [39]. These datasets were selected because they provide representative evaluation scenarios for high-resolution change detection, including complex urban backgrounds, diverse object scales, and fine-grained boundary delineation.

The WHU-CD dataset contains 480 high-resolution image pairs and mainly focuses on building changes in complex urban scenes. The LEVIR-CD+ dataset contains 985 high-resolution image pairs with a spatial resolution of 1024 × 1024 pixels, covering diverse urban scenarios involving building construction, demolition, and structural modifications. These two datasets provide complementary evaluation conditions for assessing the capability of change detection models in different spatial contexts.

To preserve spatial context and semantic integrity, the LEVIR-CD+ images were maintained at their original resolution of 1024 × 1024 pixels, while the WHU-CD images were cropped into 1024 × 1024 patches for consistency.

Both datasets were randomly divided into training, validation, and test subsets with a ratio of 8:1:1 using a fixed random seed of 42. This partition was kept unchanged throughout all the experiments. The WHU-CD dataset was divided into 384 training, 48 validation, and 48 test image pairs, while the LEVIR-CD+ dataset was divided into 788 training, 98 validation, and 99 test image pairs. The division was performed at the original image-pair level before patch generation to avoid spatial information leakage between different subsets. The same data splits were adopted for all compared methods to ensure a fair and consistent evaluation.

During training, a class-proportion-constrained random cropping strategy was used to extract 512 × 512 patches. For each candidate crop, the largest class proportion was calculated over valid labeled pixels. The candidate crop was retained if this proportion did not exceed 75% [40,41]; otherwise, a new crop location was randomly sampled from the same image pair. A maximum of 10 attempts was allowed per sample, including the initial attempt. If none of the candidates satisfied the constraint, the final candidate was retained.

The 75% threshold was adopted to reduce crops dominated by a single class while maintaining sufficient sample diversity. The purpose of this constraint was to improve the diversity of training crops and reduce the occurrence of crops containing limited discriminative information, rather than to rebalance the global proportion of change and non-change pixels. This constraint was applied only during crop selection and did not remove any original image pairs or alter the composition of the training dataset. Identical crop coordinates were applied to both temporal images and the corresponding label map. During validation and testing, each 1024 × 1024 image was divided into four non-overlapping 512 × 512 patches for inference, and the patch-level predictions were stitched together to reconstruct the full prediction map for evaluation.

In addition to the standard pixel-level evaluation metrics, a scale-dependent analysis was further conducted to evaluate the detection capability for changed regions with different spatial extents. Specifically, eight-connected components in the ground-truth change masks were identified as individual changed regions, and a COCO-inspired area-based criterion was adopted to divide them into small (<1024 pixels), medium (1024–9216 pixels), and large (≥9216 pixels) regions. Pixel-level recall was then calculated separately for each size category.

### 3.2. Evaluation Metrics

To quantitatively evaluate the performance of different change detection methods, seven commonly used metrics in remote sensing change detection studies were adopted: Precision (*P*), Recall (*R*), Overall Accuracy (*OA*), Intersection over Union (*loU*), *F*_1_ score (*F*_1_), boundary IoU (*BIoU*), and boundary F1-score (*BF*1). In addition, Runtime was also reported to provide a practical reference for computational cost. These metrics provide a comprehensive evaluation of detection accuracy, completeness, overall performance, and computational efficiency.

Precision (*P*) measures the proportion of correctly detected changed pixels among all pixels predicted as changed:

(8) $$P=\frac{\mathrm{TP}}{\mathrm{TP}+\mathrm{FP}}$$

where *TP* denotes true positives, and *FP* denotes false positives.

Recall (*R*) measures the proportion of correctly identified changed pixels among all ground-truth changed pixels:

(9) $$R=\frac{\mathrm{TP}}{\mathrm{TP}+\mathrm{FN}}$$

where *FN* denotes false negatives.

Overall Accuracy (*OA*) measures the proportion of correctly classified pixels among all pixels:

(10) $$\mathrm{OA}=\frac{\mathrm{TP}+\mathrm{TN}}{\mathrm{TP}+\mathrm{TN}+\mathrm{FP}+\mathrm{FN}}$$

where *TN* denotes true negatives.

Intersection over Union (*IoU*) measures the overlap between predicted changed regions and ground-truth changed regions:

(11) $$\mathrm{IoU}=\frac{\mathrm{TP}}{\mathrm{TP}+\mathrm{FP}+\mathrm{FN}}$$

F1-score (*F*1) is the harmonic mean of Precision and Recall and is widely used for evaluating change detection performance under class imbalance:

(12) $$F1=\frac{2\times P\times R}{P+R}$$

where *P* and *R* represent Precision and Recall, respectively.

Boundary IoU (*BIoU*) measures the overlap between the inner boundary bands of the predicted and ground-truth change regions. For an image of height H and width W, the boundary width is determined by

(13) $$d=\max\left(1,\;\mathrm{round}\left(0.02\sqrt{H^{2}+W^{2}}\right)\right)$$

The inner boundary band of a binary mask *M* is defined as *B_d_*(*M*) = *M*∖ℰ*^d^*(*M*), where ℰ*^d^* denotes *d* successive erosion operations using a 3 × 3 square structuring element. Pixels outside the image are treated as background so that boundaries of regions touching the image border are included. Let *G* and *P* denote the ground-truth and predicted binary change masks, respectively, with corresponding inner boundary bands *B_d_*(*G*) and *B_d_*(*P*). *BIoU* is defined as

(14) $$\mathrm{BIoU}=\frac{\left|B_{d}\left(G\right)\cap B_{d}\left(P\right)\right|}{\left|B_{d}\left(G\right)\cup B_{d}\left(P\right)\right|}$$

Here, |·| denotes the number of pixels in a set.

Boundary F1-score (*BF*1) [42] evaluates boundary alignment by combining boundary precision and recall within a specified spatial tolerance. Boundary pixels are extracted using the morphological gradient, defined as *∂M* = *𝒟*(*M*)∖*ℰ*(*M*), where *𝒟* and *ℰ* denote dilation and erosion with a 3 × 3 square structuring element. Pixels outside the image are treated as background, and only boundary pixels within the original image extent are retained.

A predicted boundary pixel is considered matched if its minimum Euclidean distance to a ground-truth boundary pixel does not exceed *τ* = 2 pixels. Ground-truth boundary matches are determined in the reverse direction. For a pair of masks, boundary precision *P_b_* and recall *R_b_* are defined as

(15) $$P_{b}=\frac{\left|\left\{x\in \partial P:\;\mathrm{dist}\left(x,\;\partial G\right)\leq \tau \right\}\right|}{\left|\partial P\right|}$$

(16) $$R_{b}=\frac{\left|\left\{y\in \partial G:\;\mathrm{dist}\left(y,\;\partial P\right)\leq \tau \right\}\right|}{\left|\partial G\right|}$$

(17) $$\mathrm{BF}1=\frac{2P_{b}R_{b}}{P_{b}+R_{b}}$$

Here, *∂P* and *∂G* denote the predicted and ground-truth boundary pixel sets, respectively, and $\mathrm{dist}\left(x,S\right)=\underset{s\in S}{\min}{\left\|x-s\right\|}_{2}$ denotes the minimum Euclidean distance from pixel *x* to boundary set *S*. Matching is evaluated independently in each direction without enforcing one-to-one correspondence. Runtime denotes the total inference time (in seconds) required to process all test images under the same experimental environment. Model size and computational complexity are additionally evaluated using the number of parameters (Params, M) and multiply–accumulate operations (MACs, G), respectively.

### 3.3. Experimental Setup

During training, data augmentation included random horizontal and vertical flips, Gaussian blur, and color jitter. All experiments were conducted on a workstation equipped with an Intel i9-14900KF CPU (Intel Corporation, Santa Clara, CA, USA) and an NVIDIA RTX 5090 GPU (ASUSTeK Computer Inc., Taipei, Taiwan, China). All models were trained from scratch without using pretrained weights. Training was performed using the AdamW optimizer with a weight decay of 0.01. The initial learning rate was set to 1 × 10^−4^, the batch size was set to 4, and all models were trained for 200 epochs. A linear decay learning-rate schedule was implemented using the PyTorch (2.8.0.dev20250413+cu128) LambdaLR scheduler, with the learning rate updated after each training epoch according to the predefined decay function.

To ensure a fair comparison, all compared methods were re-trained and evaluated using the same data splits, input resolution, and evaluation protocol, together with the common training settings specified above. For each baseline, the loss formulation and learning-rate scheduling strategy recommended in its original paper were retained whenever applicable. The checkpoint achieving the highest validation F1-score was selected for final evaluation. A probability threshold of 0.5 was used to generate binary change maps for all methods. Each experiment was repeated five times with different random seeds, and the mean performance and standard deviation were reported.

## 4. Results and Discussion

To comprehensively evaluate the effectiveness of SwinChange-DE, extensive experiments were conducted on the WHU-CD and LEVIR-CD+ benchmarks. The experiments include ablation studies, quantitative comparisons with representative state-of-the-art change detection methods, and qualitative visual analysis. These experiments aim to validate the effectiveness of the proposed framework in improving hierarchical spatial–semantic representation, cross-scale feature alignment, and spatial detail recovery for high-resolution remote sensing image change detection.

### 4.1. Ablation Studies

To comprehensively investigate the effectiveness of the proposed framework and its internal components, ablation experiments were conducted on the WHU-CD and LEVIR-CD+ benchmarks. The ablation analysis was performed from two complementary perspectives. First, framework-level ablation experiments were conducted based on the ChangeFormer architecture by progressively introducing the Swin-inspired hierarchical encoder, GE-SMFA, and CDDEM. Architecture-level ablation experiments were conducted on both the WHU-CD and LEVIR-CD+ datasets to evaluate the contributions of the major components to the overall framework. Second, component-wise ablation experiments were further performed within GE-SMFA and CDDEM to analyze the contributions of individual designs, including the Cross-Scale Progressive Gate, ASPP, ECA, SAM, Sobel-based edge extraction, and gated residual connection. The corresponding results for the architecture-level and component-wise ablation experiments on WHU-CD are presented in Table 1 and Table 2, respectively, while those on LEVIR-CD+ are presented in Table 3 and Table 4, respectively. The performance of each variant was evaluated using Precision (P), Recall (R), Overall Accuracy (OA), Intersection over Union (IoU), F1-score (F1), and Runtime. These experiments aim to analyze the contributions of different components to hierarchical feature representation, cross-scale feature alignment, and spatial detail recovery.

The baseline ChangeFormer achieves an IoU of 65.20% and an F1-score of 78.92%. After replacing the original encoder with the Swin Transformer, the IoU increases to 71.96%, and the F1-score improves to 83.69%. This improvement suggests that hierarchical Transformer-based feature extraction provides stronger contextual modeling capability and more effective semantic representation for complex urban scenes.

After introducing GE-SMFA, the IoU and F1-score further increase to 72.85% and 84.28%, respectively. This improvement indicates that cross-scale feature alignment helps reduce the discrepancy between shallow spatial features and deep semantic representations, enabling more reliable integration of information across different feature levels.

With the introduction of CDDEM, the complete SwinChange-DE framework achieves the highest performance, obtaining an IoU of 77.66% and an F1-score of 87.43%. Compared with the baseline model, the proposed framework improves IoU and F1-score by 12.46 and 8.51 percentage points, respectively. These results indicate that CDDEM contributes to recovering spatial information weakened during hierarchical encoding and improving boundary localization for fine-grained building change detection.

While Table 1 validates the effectiveness of the major components in the overall framework, Table 2 further investigates the contribution of individual operations within GE-SMFA and CDDEM. Removing the cross-scale progressive gating mechanism from GE-SMFA decreases IoU and F1-score by 2.85 and 1.84 percentage points, respectively, highlighting the importance of semantic guidance before feature aggregation. Within CDDEM, removing ASPP, ECA, SAM, Sobel-based edge extraction, or gated residual connection consistently reduces detection performance. These results suggest that contextual aggregation, attention refinement, edge-aware representation, and semantic-preserving residual fusion provide complementary contributions to the final framework.

A notable observation is that the effectiveness of detail recovery depends on the interaction between spatial feature distribution and semantic guidance. On the WHU-CD benchmark, which contains relatively sparse and large-scale building structures, high-resolution shallow features provide coherent boundary information. Therefore, recovered spatial details can effectively complement deep semantic representations and contribute to more complete change-region delineation.

In contrast, the improvement on the LEVIR-CD+ benchmark is relatively smaller, which may be attributed to its denser urban structures and more complex background. In such scenarios, shallow features contain richer spatial details but may also introduce high-frequency background responses. This observation highlights the importance of GE-SMFA, which performs cross-scale feature alignment before detail recovery and improves the robustness of feature interaction under different spatial distributions.

The ablation results on the LEVIR-CD+ benchmark further demonstrate the effectiveness of the proposed framework under more challenging urban environments. Compared with WHU-CD, LEVIR-CD+ contains denser building distributions and more complicated background structures, resulting in stronger interference during change localization.

As shown in Table 3, the complete SwinChange-DE achieves an IoU of 78.03% and an F1-score of 87.66%, improving the baseline by 3.49 and 2.25 percentage points, respectively. Although the improvement is smaller than that observed on WHU-CD, the complete model achieves the best overall performance among the evaluated configurations, suggesting that the proposed components jointly contribute to improved change representation.

While Table 3 evaluates the contribution of the major components in the framework, Table 4 further analyzes the contribution of individual operations within GE-SMFA and CDDEM under complex urban scenarios. Removing the cross-scale progressive gating mechanism, ASPP, ECA, SAM, Sobel-based edge extraction, or gated residual connection results in slight performance degradation, although the magnitude of change is smaller than that observed on WHU-CD. These results suggest that these components provide incremental and complementary improvements for change localization in complex backgrounds.

Overall, the ablation results indicate that SwinChange-DE improves change detection performance through a progressive optimization process, from hierarchical spatial–semantic representation learning to cross-scale feature alignment and spatial detail recovery. The complementary interaction among these components enables more accurate localization of building changes while maintaining structural consistency.

### 4.2. Comparative Experiments

To further evaluate the effectiveness of SwinChange-DE, comparative experiments were conducted on the WHU-CD and LEVIR-CD+ benchmarks. SwinChange-DE was compared with seven representative and widely adopted change detection methods, including ChangeFormer, HANet [43], DTCDSCN [44], BiT [45], ScratchFormer [46], ICIFNET [47], and BIFA [48]. These methods cover convolution-based, Transformer-based, and hybrid feature interaction strategies. All compared methods were re-trained and evaluated using the same data splits, input resolution, training strategy, and evaluation protocol to ensure a fair comparison.

As reported in Table 5, SwinChange-DE achieves the best overall performance on the WHU-CD benchmark, obtaining an IoU of 77.66% and an F1-score of 87.43%. Compared with existing state-of-the-art methods, SwinChange-DE consistently improves change detection accuracy, demonstrating its effectiveness in handling complex urban scenes with high-resolution spatial information.

Compared with ChangeFormer, which serves as the baseline framework, SwinChange-DE improves IoU and F1-score by 12.46% and 8.51%, respectively. This improvement verifies the effectiveness of the proposed modifications beyond the original hierarchical Transformer framework. Specifically, the Swin-inspired hierarchical encoder provides stronger hierarchical spatial–semantic representation through efficient contextual modeling, while GE-SMFA further reduces the discrepancy between shallow spatial features and deep semantic representations through cross-scale feature alignment.

Furthermore, CDDEM contributes to the recovery of spatial structures that are weakened during hierarchical feature extraction. By integrating refined shallow features with deep semantic representations, CDDEM improves boundary localization and structural completeness, which is particularly beneficial for building-change detection with complex object shapes.

Compared with other advanced approaches, including BiT, ScratchFormer, ICIF-NET, and BIFA, SwinChange-DE achieves higher IoU and F1-score performance. This indicates that simply improving contextual modeling or feature interaction is insufficient for fine-grained change detection, and effective preservation of spatial details is also critical for accurately delineating changed regions.

In terms of computational efficiency, SwinChange-DE introduces additional computational overhead due to the proposed feature alignment and detail recovery operations. Compared with ChangeFormer, SwinChange-DE requires longer inference time, reflecting the trade-off between improved detection performance and computational cost.

As shown in Table 6, SwinChange-DE achieves the highest detection performance on the LEVIR-CD+ benchmark, with an IoU of 78.03% and an F1-score of 87.66%. Compared with ChangeFormer, SwinChange-DE improves IoU and F1-score by 3.49% and 2.25%, respectively, demonstrating the effectiveness of the proposed detail-preserving hierarchical design.

Compared with other representative methods, SwinChange-DE achieves consistent improvements across multiple evaluation metrics. These results indicate that the proposed framework can effectively address the challenges associated with densely distributed buildings and complex urban backgrounds in high-resolution remote sensing imagery.

The performance improvement mainly benefits from the complementary functions of GE-SMFA and CDDEM. GE-SMFA performs cross-scale feature alignment to improve the consistency between spatial details and semantic information, reducing irrelevant responses caused by heterogeneous feature distributions. Meanwhile, CDDEM focuses on recovering fine spatial structures and improving boundary delineation during decoding, which is important for detecting subtle building changes.

Although the improvement over the strongest competing method is smaller on LEVIR-CD+ than on WHU-CD, SwinChange-DE achieves competitive performance among the evaluated methods on both benchmarks. The performance margin over some competitive approaches on LEVIR-CD+ is relatively limited, suggesting that the advantage of the proposed framework is reflected not only in region-level metrics but also in boundary preservation and structural consistency.

Overall, the quantitative comparison results demonstrate that SwinChange-DE effectively improves high-resolution remote sensing image change detection by jointly optimizing hierarchical representation learning, cross-scale feature alignment, and spatial detail recovery. The proposed framework not only enhances numerical accuracy but also provides more reliable structural representation for fine-grained change localization.

### 4.3. Boundary Detection Performance

To quantitatively assess the ability of SwinChange-DE to delineate building boundaries accurately, we further evaluate its performance using Boundary IoU (BIoU) and boundary F1-score (BF1) on the WHU-CD and LEVIR-CD+ datasets. While region-based metrics such as IoU and F1-score reflect the overall accuracy of detected change regions, these boundary-focused metrics provide a more targeted assessment of the agreement between predicted and ground-truth contours. This evaluation complements the preceding quantitative comparisons by examining whether improvements in overall detection performance are accompanied by more precise delineation of building-change boundaries.

As shown in Table 7, SwinChange-DE achieves the highest mean BIoU and BF1 scores on both the WHU-CD and LEVIR-CD+ datasets, suggesting improved boundary-related prediction performance. On WHU-CD, SwinChange-DE obtains a BIoU of 68.74% and a BF1 of 62.19%, exceeding the ChangeFormer baseline by 11.95 and 21.59 percentage points, respectively. Compared with the strongest competing results, it improves BIoU by 0.63 percentage points over HANet and BIFA, and increases BF1 by 5.62 percentage points over BIFA.

The improvement in BF1 provides additional evidence of enhanced boundary alignment under the prescribed matching tolerance, complementing the boundary-band overlap measured by BIoU. On LEVIR-CD+, SwinChange-DE achieves a BIoU of 77.45% and a BF1 of 83.74%, improving upon ChangeFormer by 3.43 and 3.74 percentage points, respectively. It also surpasses ScratchFormer, the strongest competing method on both boundary metrics, by 0.40 and 0.32 percentage points. The improvements observed on both datasets complement the region-level results presented in Table 5 and Table 6, further supporting the effectiveness of the proposed framework in preserving boundary details. These findings are consistent with the design objective of SwinChange-DE, which integrates hierarchical feature learning, cross-scale feature interaction, and semantic-guided detail recovery to improve the balance between semantic representation and spatial detail preservation.

### 4.4. Qualitative Analysis

**Figure 4:**
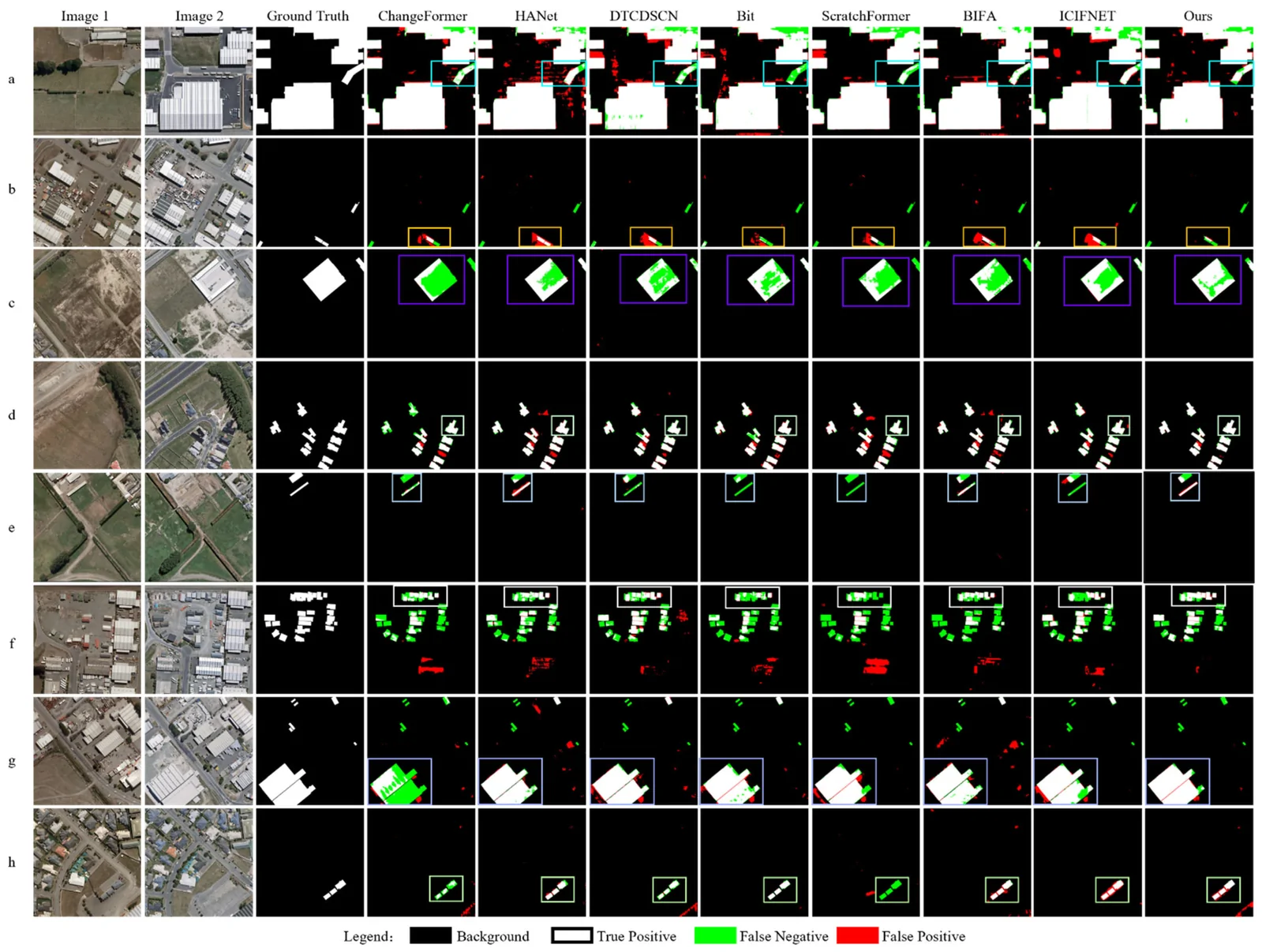
Qualitative comparison of different change detection methods on the WHU-CD dataset. (**a**) Comparison of missed detections for buildings; (**b**) Comparison of missed and false detections for small buildings; (**c**) Comparison of missed detections at the centers of large buildings; (**d**) Comparison of edge sharpness for small buildings; (**e**) Comparison of missed detections for small buildings; (**f**) Comparison of missed detections for clustered buildings; (**g**) Comparison of edge sharpness for large buildings; (**h**) Comparison of missed detections for clustered small buildings.

**Figure 5:**
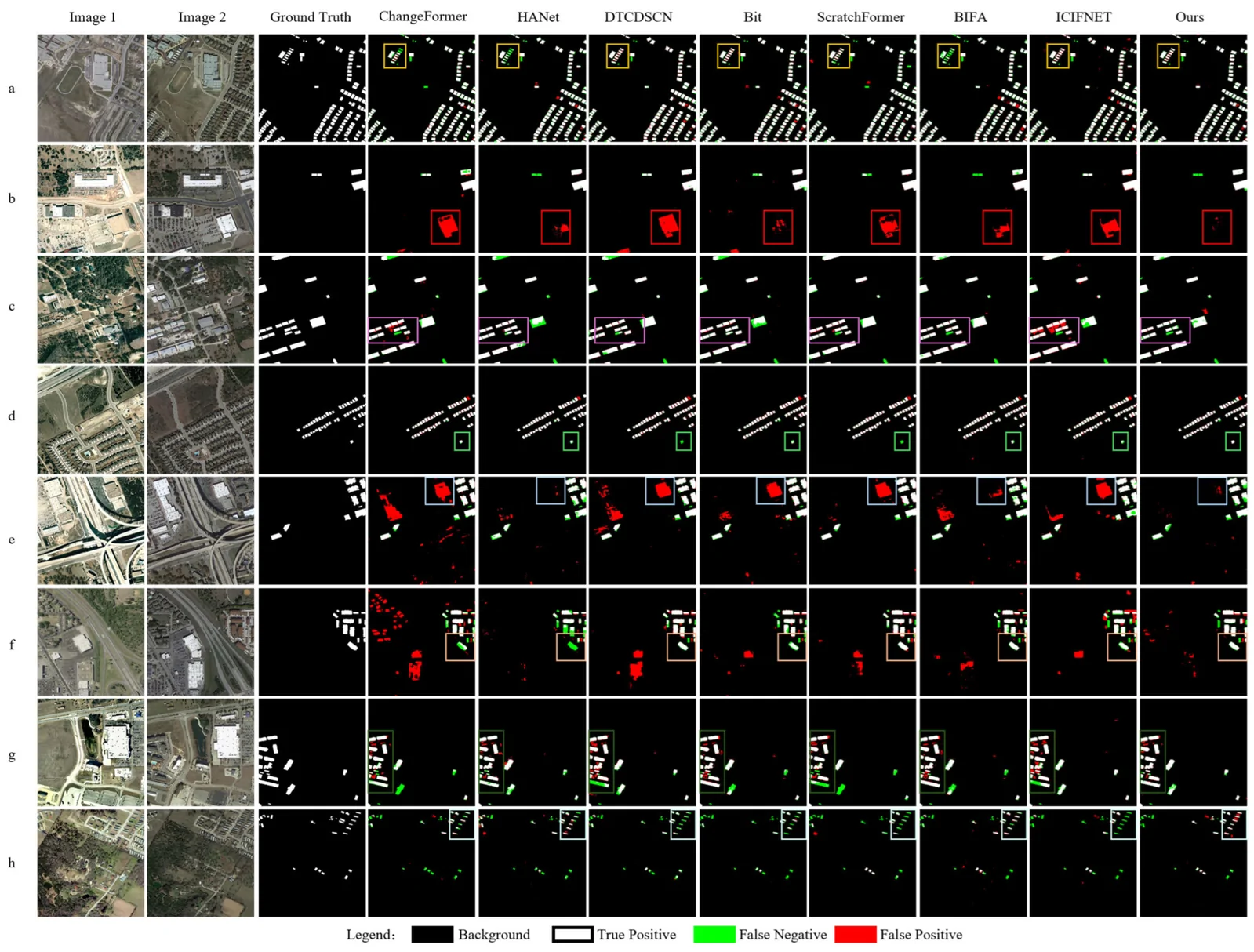
Qualitative comparison of different change detection methods on the LEVIR-CD+ dataset. (**a**) Comparison of missed and false detections for clustered small buildings; (**b**) Comparison of false detections in background regions; (**c**) Comparison of missed detections, false detections, and edge sharpness for clustered small buildings; (**d**) Comparison of missed detections for small buildings; (**e**) Comparison of false detections in background regions; (**f**) Comparison of missed detections and edge sharpness for small buildings; (**g**) Comparison of missed detections and edge sharpness for clustered small buildings; (**h**) Comparison of missed detections for clustered small buildings.

To further analyze the visual performance of different change detection methods, qualitative comparisons were conducted on representative samples from the WHU-CD and LEVIR-CD+ datasets. The visualization results are presented in Figure 4 and Figure 5. These examples are selected to evaluate the capability of different methods in preserving object boundaries, detecting small-scale changes, and suppressing false-positive responses under complex urban environments.

As shown in Figure 4, the compared methods exhibit different levels of accuracy in identifying changed regions. Some approaches generate incomplete change maps or fragmented boundaries, especially in areas with irregular building structures and complex surrounding backgrounds. These errors indicate that insufficient spatial detail preservation may lead to inaccurate localization of changed objects.

Transformer-based methods generally provide improved contextual modeling capability; however, the progressive spatial reduction during hierarchical feature extraction may still weaken fine spatial structures. As a result, some compared methods produce blurred boundaries or miss subtle changed regions.

In contrast, SwinChange-DE generates more complete and structurally consistent change regions. The improved visual quality benefits from the complementary functions of GE-SMFA and CDDEM. GE-SMFA enhances cross-scale feature alignment by reducing the discrepancy between shallow spatial features and deep semantic representations, while CDDEM recovers spatial details lost during hierarchical encoding and improves boundary refinement during decoding. Therefore, SwinChange-DE is able to preserve building contours and detect fine-grained changes more accurately.

The qualitative results on the LEVIR-CD+ dataset further demonstrate the advantages of SwinChange-DE in challenging urban scenarios. Due to the dense distribution of buildings, complex background textures, and small-scale structural variations, LEVIR-CD+ presents greater difficulties for accurate change localization.

As illustrated in Figure 5, several compared methods suffer from false detections and incomplete change regions, particularly in areas containing densely distributed buildings or complex spatial patterns. These limitations suggest that directly relying on semantic representation or spatial features alone is insufficient for fine-grained change detection.

Compared with other methods, SwinChange-DE produces more continuous change regions with improved geometric consistency and fewer spurious responses. This improvement can be attributed to the hierarchical design of the proposed framework: the Swin-inspired hierarchical encoder provides robust spatial–semantic representation, GE-SMFA establishes effective cross-scale feature alignment, and CDDEM enhances spatial detail recovery and boundary preservation.

Overall, the qualitative analysis confirms that SwinChange-DE not only improves quantitative accuracy but also provides more reliable structural representation for high-resolution remote sensing image change detection. The generated change maps demonstrate improved boundary continuity, reduced false alarms, and enhanced capability for detecting subtle spatial variations.

## 5. Conclusions

This paper proposes SwinChange-DE, a detail-preserving hierarchical Transformer framework for high-resolution remote sensing image change detection. The framework is designed to address the challenges of insufficient multi-scale representation, spatial detail degradation, and inaccurate boundary localization in complex remote sensing scenes. By integrating a Swin-inspired hierarchical encoder, GE-SMFA, and CDDEM, SwinChange-DE establishes a semantic-guided feature learning framework that jointly considers contextual representation, cross-scale alignment, and detail recovery.

Extensive experiments on the WHU-CD and LEVIR-CD+ benchmarks demonstrate the effectiveness of the proposed framework. Ablation studies confirm that the three components provide complementary contributions: the Swin-inspired encoder improves multi-level representation learning, GE-SMFA enhances cross-scale feature alignment, and CDDEM facilitates spatial detail recovery during decoding. Comparative experiments further show that SwinChange-DE achieves improved IoU and F1-score performance compared with representative change detection methods, while additional boundary evaluations indicate improved boundary delineation capability.

Overall, the results suggest that the performance gains of SwinChange-DE arise from the collaborative optimization of hierarchical representation learning, semantic-guided feature refinement, and detail-preserving decoding. Rather than enhancing a single processing stage, the proposed framework integrates semantic discrimination and spatial structural preservation within an end-to-end architecture, providing an effective solution for high-resolution remote sensing image change detection.

Despite its improved performance, SwinChange-DE still has several limitations. The incorporation of multi-scale feature interaction and detail recovery modules introduces additional computational cost compared with some lightweight approaches. In addition, qualitative analysis reveals remaining false-positive responses in complex background regions and incomplete detection within some large, changed buildings. These observations indicate that improving background discrimination and maintaining spatial consistency within heterogeneous change regions remain important challenges. Furthermore, the current evaluation mainly focuses on optical building-change datasets, and the generalization capability of the framework across diverse land-cover categories, geographic regions, and multi-source remote sensing data requires further investigation.

Future studies may explore model compression strategies, such as structured channel pruning and knowledge distillation, to reduce computational overhead while preserving detection performance. In addition, more effective hard-negative learning strategies and improved detail recovery mechanisms may further enhance background suppression and the completeness of predicted change regions.

## Figures and Tables

**Table 1:** Framework-level ablation results of SwinChange-DE on the WHU-CD benchmark.

Swin Transformer	GE-SMFA	CDDEM	P	R	OA	IoU	F1-Score	Runtime(s)
			83.82 ± 3.54	74.64 ± 0.60	98.44 ± 0.13	65.20 ± 1.77	78.92 ± 1.29	4.55 ± 0.03
√			86.09 ± 1.31	81.44 ± 0.25	98.76 ± 0.05	71.96 ± 0.83	83.69 ± 0.57	4.34 ± 0.05
√	√		83.77 ± 2.54	84.84 ± 0.47	98.77 ± 0.10	72.85 ± 1.55	84.28 ± 1.03	5.60 ± 0.01
√		√	86.99 ± 1.07	82.67 ± 1.03	98.84 ± 0.05	73.57 ± 0.99	84.77 ± 0.66	5.27 ± 0.01
	√		82.65 ± 0.87	86.10 ± 0.28	98.75 ± 0.04	72.92 ± 0.55	84.34 ± 0.37	5.71 ± 0.01
	√	√	88.50 ± 1.15	84.49 ± 0.32	98.97 ± 0.06	76.14 ± 1.08	86.45 ± 0.70	6.70 ± 0.05
		√	84.34 ± 0.39	75.84 ± 1.42	98.65 ± 0.17	66.46 ± 0.85	79.85 ± 0.61	5.48 ± 0.06
√	√	√	88.76 ± 0.45	86.15 ± 0.98	99.03 ± 0.02	77.66 ± 0.66	87.43 ± 0.42	6.60 ± 0.01

“√” denotes that the corresponding component (Swin Transformer, GE-SMFA or CDDEM) is incorporated into the network.

**Table 2:** Component-wise ablation analysis of GE-SMFA and CDDEM on the WHU-CD benchmark.

Model	P	R	OA	IoU	F1-Score	Runtime(s)
SwinChange-DE	88.76 ± 0.45	86.15 ± 0.98	99.03 ± 0.02	77.66 ± 0.66	87.43 ± 0.42	6.60 ± 0.01
Without Cross-Scale Progressive Gate	87.21 ± 0.81	84.05 ± 1.78	98.90 ± 0.06	74.81 ± 1.38	85.59 ± 0.90	6.41 ± 0.32
Without ASPP	85.00 ± 2.01	85.42 ± 0.74	98.84 ± 0.11	74.23 ± 1.89	85.20 ± 1.25	5.52 ± 0.01
Without ECA–SAM	88.72 ± 0.76	84.31 ± 0.77	98.97 ± 0.02	76.14 ± 0.38	86.45 ± 0.25	6.41 ± 0.25
Without Sobel-based edge extraction	87.18 ± 1.07	85.31 ± 0.50	98.94 ± 0.04	75.79 ± 0.57	86.23 ± 0.37	6.13 ± 0.22
Without ECA	86.24 ± 1.82	85.06 ± 1.07	98.89 ± 0.07	74.87 ± 1.23	85.63 ± 0.81	6.10 ± 0.03
Without SAM	87.77 ± 0.13	83.46 ± 0.77	98.90 ± 0.03	74.76 ± 0.69	85.55 ± 0.45	6.04 ± 0.04
Without Gated Residual	88.00 ± 0.84	84.29 ± 0.59	98.94 ± 0.02	75.59 ± 0.39	86.10 ± 0.25	6.22 ± 0.27

**Table 3:** Framework-level ablation results of SwinChange-DE on the LEVIR-CD+ benchmark.

Swin Transformer	GE-SMFA	CDDEM	P	R	OA	IoU	F1-Score	Runtime(s)
			85.63 ± 0.71	85.21 ± 0.42	98.72 ± 0.02	74.54 ± 0.22	85.41 ± 0.14	8.92 ± 0.08
√			87.88 ± 0.07	85.95 ± 0.28	98.86 ± 0.01	76.83 ± 0.20	86.90 ± 0.13	8.34 ± 0.01
√	√		88.47 ± 0.39	85.75 ± 0.62	98.89 ± 0.01	77.12 ± 0.23	87.09 ± 0.15	11.08 ± 0.03
√		√	86.19 ± 0.99	86.36 ± 0.76	98.80 ± 0.02	75.85 ± 0.28	86.27 ± 0.18	10.29 ± 0.08
	√		87.32 ± 0.38	86.07 ± 0.49	98.84 ± 0.02	76.50 ± 0.28	86.69 ± 0.18	11.27 ± 0.04
	√	√	88.11 ± 0.54	86.87 ± 0.38	98.91 ± 0.01	77.75 ± 0.12	87.48 ± 0.07	13.24 ± 0.09
		√	86.13 ± 0.07	84.49 ± 0.26	98.72 ± 0.01	74.37 ± 0.16	85.30 ± 0.11	10.74 ± 0.01
√	√	√	88.14 ± 0.15	87.19 ± 0.33	98.93 ± 0.01	78.03 ± 0.26	87.66 ± 0.16	13.02 ± 0.06

“√” denotes that the corresponding component (Swin Transformer, GE-SMFA or CDDEM) is incorporated into the network.

**Table 4:** Component-wise ablation analysis of GE-SMFA and CDDEM on the LEVIR-CD+ benchmark.

Model	P	R	OA	IoU	F1-Score	Runtime(s)
SwinChange-DE	88.14 ± 0.15	87.19 ± 0.33	98.93 ± 0.01	78.03 ± 0.26	87.66 ± 0.16	13.02 ± 0.06
Without Cross-Scale Progressive Gate	88.26 ± 0.08	86.40 ± 0.43	98.90 ± 0.02	77.49 ± 0.31	87.32 ± 0.20	12.83 ± 0.07
Without ASPP	88.89 ± 0.85	85.69 ± 0.81	98.90 ± 0.01	77.39 ± 0.10	87.26 ± 0.07	11.48 ± 0.02
Without ECA–SAM	88.77 ± 0.12	85.87 ± 0.18	98.90 ± 0.01	77.46 ± 0.20	87.30 ± 0.13	12.90 ± 0.04
Without Sobel-based edge extraction	88.07 ± 0.55	86.57 ± 0.40	98.90 ± 0.02	77.48 ± 0.29	87.31 ± 0.18	12.09 ± 0.02
Without ECA	88.66 ± 0.13	86.28 ± 0.25	98.91 ± 0.01	77.70 ± 0.21	87.46 ± 0.13	12.90 ± 0.26
Without SAM	87.35 + 0.31	87.44 + 0.43	98.90 + 0.01	77.61 + 0.13	87.39 + 0.08	12.47 + 0.08
Without Gated Residual	87.86 ± 0.68	86.53 ± 0.72	98.89 ± 0.01	77.28 ± 0.20	87.18 ± 0.13	12.67 ± 0.24

**Table 5:** Quantitative comparison with state-of-the-art methods on the WHU-CD benchmark.

Method	P	R	OA	IoU	F1-Score	Runtime(s)
ChangeFormer	83.82 ± 3.54	74.64 ± 0.60	98.44 ± 0.13	65.20 ± 1.77	78.92 ± 1.29	4.55 ± 0.03
HANet	87.31 ± 3.08	85.76 ± 1.20	98.95 ± 0.10	76.22 ± 1.61	86.50 ± 1.04	4.83 ± 0.01
DTCDSCN	84.02 ± 1.57	83.95 ± 1.14	98.75 ± 0.09	72.40 ± 1.72	83.98 ± 1.16	1.34 ± 0.03
BiT	86.87 ± 0.98	82.11 ± 0.48	98.82 ± 0.03	73.04 ± 0.48	84.42 ± 0.32	1.83 ± 0.02
ScratchFormer	82.30 ± 1.21	77.71 ± 1.86	98.48 ± 0.05	66.56 ± 1.14	79.92 ± 0.82	6.05 ± 0.08
BIFA	89.00 ± 1.21	83.24 ± 1.69	98.95 ± 0.02	75.44 ± 0.60	86.00 ± 0.39	7.85 ± 0.12
ICIF-NET	87.71 ± 0.88	83.46 ± 0.29	98.90 ± 0.03	74.71 ± 0.41	85.52 ± 0.27	2.96 ± 0.03
SwinChange-DE	88.76 ± 0.45	86.15 ± 0.98	99.03 ± 0.02	77.66 ± 0.66	87.43 ± 0.42	6.60 ± 0.01

**Table 6:** Quantitative comparison with state-of-the-art methods on the LEVIR-CD+ benchmark.

Method	P	R	OA	IoU	F1-Score	Runtime(s)
ChangeFormer	85.63 ± 0.71	85.21 ± 0.42	98.72 ± 0.02	74.54 ± 0.22	85.41 ± 0.14	8.92 ± 0.08
HANet	87.69 ± 1.76	85.31 ± 1.31	98.83 ± 0.06	76.15 ± 0.82	86.46 ± 0.53	9.62 ± 0.02
DTCDSCN	86.38 ± 1.00	86.88 ± 0.78	98.82 ± 0.02	76.39 ± 0.22	86.62 ± 0.14	2.39 ± 0.08
Bit	87.88 ± 0.23	83.66 ± 0.55	98.78 ± 0.03	75.00 ± 0.60	85.72 ± 0.39	3.36 ± 0.04
ScratchFormer	88.23 ± 0.34	86.62 ± 1.01	98.91 ± 0.03	77.64 ± 0.67	87.41 ± 0.42	11.77 ± 0.04
BIFA	87.47 ± 0.64	86.77 ± 0.36	98.88 ± 0.02	77.18 ± 0.23	87.12 ± 0.15	15.86 ± 0.01
ICIF-NET	84.67 ± 0.50	85.70 ± 0.80	98.69 ± 0.01	74.18 ± 0.32	85.17 ± 0.21	5.70 ± 0.04
SwinChange-DE	88.14 ± 0.15	87.19 ± 0.33	98.93 ± 0.01	78.03 ± 0.26	87.66 ± 0.16	13.02 ± 0.06

**Table 7:** Comparison of boundary detection performance and computational complexity on the WHU-CD and LEVIR-CD+ datasets.

Method	WHU-CD		LEVIR-CD+		Param(M)	FLOPs
	BIoU	BF1	BIoU	BF1		
ChangeFormer	56.79 ± 3.43	40.60 ± 5.46	74.02 ± 0.15	80.00 ± 0.40	41.03	4478.81
HANet	68.11 ± 1.54	50.28 ± 1.86	75.39 ± 0.78	79.72 ± 1.40	2.61	775.44
DTCDSCN	63.05 ± 2.67	44.05 ± 1.52	75.85 ± 0.13	81.12 ± 0.10	41.07	366.35
Bit	61.48 ± 0.88	46.82 ± 0.73	74.14 ± 0.59	79.50 ± 0.70	12.40	346.17
ScratchFormer	57.79 ± 1.66	44.86 ± 2.25	77.05 ± 0.70	83.42 ± 0.70	37.00	4232.03
BIFA	68.11 ± 0.34	56.57 ± 0.83	76.48 ± 0.19	81.24 ± 0.31	9.87	1705.56
ICIF-NET	65.89 ± 0.35	52.16 ± 2.03	73.58 ± 0.35	77.56 ± 0.41	25.83	838.77
SwinChange-DE	68.74 ± 0.70	62.19 ± 0.31	77.45 ± 0.28	83.74 ± 0.09	45.14	6039.01

## Data Availability

The publicly available datasets LEVIR-CD+ and WHU-CD used in this study can be accessed via their official repositories. Additional processed experimental data supporting the findings are available from the corresponding author upon reasonable request.
